# RETRACTION: LncRNA SNHG4 Promotes Tumour Growth by Sponging miR‐224‐3p and Predicts Poor Survival and Recurrence in Human Osteosarcoma

**DOI:** 10.1111/cpr.70217

**Published:** 2026-05-14

## Abstract

**RETRACTION**: XuR. , FengF. , YuX. , LiuZ. , and LaoL. , “LncRNA SNHG4 Promotes Tumour Growth by Sponging miR‐224‐3p and Predicts Poor Survival and Recurrence in Human Osteosarcoma,” Cell Proliferation 51, no. 6 (2018): e12515, 10.1111/cpr.12515.30152090 PMC6528889

The above article, published online on 28 August 2018 in Wiley Online Library (http://onlinelibrary.wiley.com/), has been retracted by agreement between the journal Editor‐in‐Chief, Qi Zhou; and John Wiley & Sons Ltd. Concerns were raised by a third party regarding duplicated images in multiple figures. An investigation by the publisher found apparent duplications within Figures 3C and 5B, as well as images reused in or taken from other articles by different authors: 2E in Li et al. 2016 (https://doi.org/10.2147/JPR.S118581) and Guo et al. 2019 (https://doi.org/10.1155/2019/4390839); 5F in Zhang et al. 2018 (https://doi.org/10.1590/1414‐431X20187439) and Zhu et al. 2019 (https://doi.org/10.18632/aging.102600); and 5B in Chen et al. 2019 (https://doi.org/10.1177/205873841882074) and Zhang et al. 2019 (https://doi.org/10.1007/s10120‐019‐01018‐7). Due to the extent of these apparent duplications, the editor has lost confidence in the results reported, and therefore the article must be retracted. Corresponding author Lifeng Lao agrees with this decision. The other authors did not respond to the publisher's notice of retraction.

**RETRACTION**: R. Xu , F. Feng , X. Yu , Z. Liu , and L. Lao , “LncRNA SNHG4 Promotes Tumour Growth by Sponging miR‐224‐3p and Predicts Poor Survival and Recurrence in Human Osteosarcoma,” Cell Proliferation 51, no. 6 (2018): e12515, 10.1111/cpr.12515.30152090 PMC6528889

The above article, published online on 28 August 2018 in Wiley Online Library (http://onlinelibrary.wiley.com/), has been retracted by agreement between the journal Editor‐in‐Chief, Qi Zhou; and John Wiley & Sons Ltd. Concerns were raised by a third party regarding duplicated images in multiple figures. An investigation by the publisher found apparent duplications within Figures 3C and 5B, as well as images reused in or taken from other articles by different authors: 2E in Li et al. 2016 (https://doi.org/10.2147/JPR.S118581) and Guo et al. 2019 (https://doi.org/10.1155/2019/4390839); 5F in Zhang et al. 2018 (https://doi.org/10.1590/1414‐431X20187439) and Zhu et al. 2019 (https://doi.org/10.18632/aging.102600); and 5B in Chen et al. 2019 (https://doi.org/10.1177/205873841882074) and Zhang et al. 2019 (https://doi.org/10.1007/s10120‐019‐01018‐7). Due to the extent of these apparent duplications, the editor has lost confidence in the results reported, and therefore the article must be retracted. Corresponding author Lifeng Lao agrees with this decision. The other authors did not respond to the publisher's notice of retraction.

